# Morphological Features and HIF1-Dependent Processes in the Brain of Progeny of Female Rats Exposed to Maternal Hypoxia

**DOI:** 10.3390/ijms27083421

**Published:** 2026-04-10

**Authors:** Sofiya Potapova, Elizaveta Zugan, Yan Isakov, Ekaterina Tyulkova, Oleg Vetrovoy

**Affiliations:** Laboratory of Regulation of Brain Neuronal Functions, Pavlov Institute of Physiology, Russian Academy of Sciences, Makarova Emb. 6, 199034 Saint-Petersburg, Russia; sofiya-potapova@mail.ru (S.P.); etyulkova@yandex.ru (E.T.)

**Keywords:** maternal hypoxia, transgenerational effect, brain morphology, HIF1

## Abstract

Fetal hypoxia and maternal stress during pregnancy are major risk factors for neurological disorders. The effects of maternal hypoxia may be transmitted to the next generation through persistent alterations in maternal endocrine and metabolic regulation. In this study, using immunohistochemistry, quantitative RT-PCR, and Western blotting, we assessed morphological features and HIF1-dependent processes in the fetal and adult brains of progeny of female rats exposed to maternal hypoxia (PMH). We identified a delay in progenitor cell differentiation into neurons at embryonic day 14, a decreased number of neurons in the hippocampus, an increased number of astrocytes in the prefrontal cortex, and a decreased number of astrocytes in the raphe nuclei of the PMH rats. However, no significant changes were observed in HIF1α protein levels or in the protein levels of HIF1-dependent gene products in the examined brain structures. Thus, the transgenerational effect of maternal hypoxia is manifested as structural disturbances of brain development but is not accompanied by changes in HIF1-dependent metabolism.

## 1. Introduction

Brain development is shaped not only by genetic programs but also by environmental influences acting during sensitive prenatal and early postnatal periods [1]. Among adverse intrauterine factors, hypoxia is of particular importance because it is a common complication of pregnancy and is strongly associated with adverse neurodevelopmental outcomes [2,3,4,5,6]. Clinical and experimental evidence indicates that prenatal hypoxia can disrupt neuronal differentiation, synaptic maturation, neuroplasticity, and later cognitive and emotional function [1,2,3,4,5,6].

In the present study, we focus specifically on maternal hypoxia (MH) as the experimental model. This model is especially relevant because it combines reduced oxygen availability with the maternal physiological and glucocorticoid response to hypoxic stress [3,4,5], as well as placental adaptation to this challenge. Accordingly, MH is suitable for testing not only direct effects on fetal development but also whether hypoxic pregnancy can alter developmental trajectories in the next generation.

The fetal brain is especially vulnerable to oxygen deficiency because of its high metabolic demand and the tight coupling between oxygen availability and developmental processes such as proliferation, differentiation, axonal growth, and synaptogenesis [1,2,3]. In rodents, late gestation represents a critical period for maturation of forebrain and limbic structures, including the hippocampus and cortex [5,6,7]. Consistent with this developmental vulnerability, prenatal hypoxia has been shown to induce long-lasting alterations in neuroplasticity, stress responsivity, and behavior in the offspring [8,9,10,11].

Importantly, the consequences of hypoxic pregnancy may extend beyond the directly exposed fetus. Stable changes in maternal physiology, endocrine signaling, placental function, and epigenetic regulation provide plausible routes by which exposure in one generation may influence fetal development in the next [11,12]. Within this framework, the placenta should be considered not as a passive transport barrier but as an active regulator of fetal neurodevelopment that integrates oxygen supply, nutrient transfer, glucocorticoid exposure, inflammatory signals, and endocrine cues [13,14].

A central molecular candidate in this process is the hypoxia-inducible factor 1 (HIF1) pathway. HIF1 coordinates transcriptional responses to reduced oxygen availability and regulates genes involved in angiogenesis, glucose metabolism, oxygen homeostasis, and cell survival [12,15]. At the same time, persistent structural and cellular changes in the absence of sustained HIF1-related activation would suggest that the long-term effects of MH are mediated predominantly by developmental reprogramming rather than by ongoing hypoxia-responsive signaling. This rationale underlies our combined assessment of HIF1α in the placenta and fetal brain together with morphological and cellular endpoints [10,11,12,13,14].

In our previous studies, we showed that hypoxic stress during pregnancy induces persistent changes in chromatin-related epigenetic regulation in the offspring brain, alters HIF1-related signaling and glucose metabolism, and reduces glucocorticoid sensitivity in extrahypothalamic brain structures through decreased glucocorticoid receptor expression and reduced glucocorticoid-dependent transcriptional activity [9,15,16,17]. These findings support the concept that MH engages interconnected hypoxic, endocrine, and epigenetic mechanisms. However, it remains unclear whether such mechanisms are associated with altered fetal brain growth and with persistent region-specific changes in brain organization in the next generation.

In particular, limited information is available on whether maternal hypoxia (MH) in the previous generation affects fetal brain growth and neurodevelopmental trajectories in the next generation, placental versus fetal brain hypoxia-responsive signaling during gestation, and region-specific neuronal and astroglial organization in the adult brain. To address this question, we examined brain regions that belong to stress-, emotion-, and motivation-related circuits but differ in developmental timing and functional specialization. This question is especially relevant for structures involved in cognition, emotional regulation, and motivated behavior, including the hippocampus (HPC), medial prefrontal cortex (PFC), amygdala (AMG), nucleus accumbens (NAcc), ventral tegmental area (VTA), and dorsal raphe nucleus (RN), all of which are known to be sensitive to developmental stress and hypoxic insults. The HPC and PFC were selected because they are key structures for cognition, executive regulation, and glucocorticoid-sensitive plasticity. The AMG, NAcc, VTA, and RN were included because they form interconnected limbic and monoaminergic networks involved in emotional regulation, reinforcement, and stress-related behavioral responses. Analysis of this set of regions allowed us to test whether the effects of MH are generalized or region-selective and whether they preferentially involve higher-order limbic-cortical and monoaminergic pathways [4,5,6,9,10,11]. If fetal hypoxia and the maternal stress response to hypoxia affect the development of individual brain structures during specific stages of embryogenesis, then the transgenerational effects of prenatal hypoxia may be shaped by persistent disturbances in maternal signaling pathways acting on the fetus throughout pregnancy. This consideration, in turn, provides a rationale for examining brain structures that differ substantially in their developmental timing.

Therefore, the aim of the present study was to evaluate the effects of maternal hypoxia on fetal brain development and adult brain structural organization in the next generation, with special attention to placental and brain HIF1-related signaling. Specifically, we assessed fetal brain growth and neurodevelopmental markers (proliferating cells and differentiated neurons), HIF1α protein content in the fetal placenta and fetal brain, and, in adult second-generation offspring, region-specific neuronal and astrocytic parameters as well as Hif1α expression and selected HIF1-dependent metabolic transcripts/proteins in limbic and monoaminergic brain structures.

## 2. Results

### 2.1. Consequences of Maternal Hypoxia on the Next Generation Fetal Brain Development

**Figure 1:**
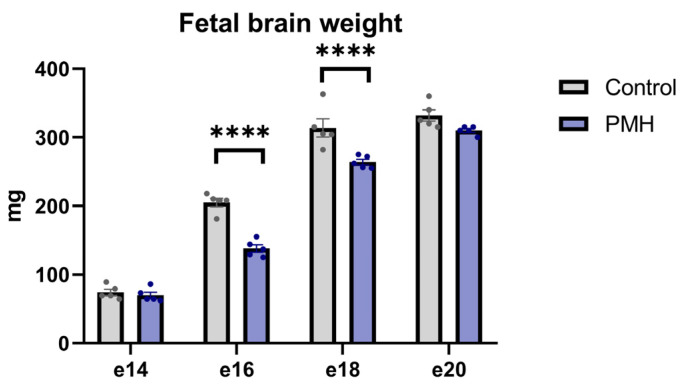
Fetal brain weight in the Control and PMH groups at embryonic days e14, e16, e18, and e20. Fetal brain weight was analyzed using an ordinary two-way ANOVA with Group (Control vs. PMH) and Time (e14, e16, e18, e20) as factors. The analysis revealed significant effects of Time (F(3,32) = 549.2, *p* < 0.0001) and Group (F(1,32) = 53.92, *p* < 0.0001), as well as a significant Group × Time interaction (F(3,32) = 8.291, *p* = 0.0003). Sidak’s multiple comparisons test (Control vs. PMH within each embryonic day) showed significantly lower fetal brain weight in the PMH group at e16 (adjusted *p* < 0.0001) and e18 (adjusted *p* < 0.0001), while differences were not significant at e14 or e20. **** adjusted *p* < 0.0001. Bars represent mean ± SEM; dots indicate individual values, n = 5 per group.

Fetal brain weight in the Control and progeny of the maternal hypoxic female (PMH) rats was assessed on embryonic days e14, e16, e18, and e20 (Figure 1). No significant difference in fetal brain weight was observed at e14 and e20. However, fetal brain weight in the PMH group was significantly lower than in the Control group at e16 (PMH vs. Control, *p* < 0.0001, Sidak’s multiple comparisons test) and e18 (PMH vs. Control, *p* < 0.0001, Sidak’s multiple comparisons test).

**Figure 2:**
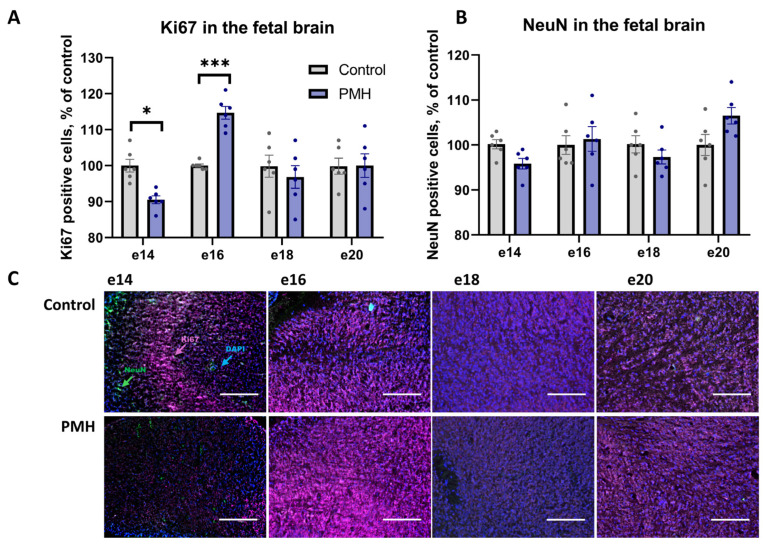
Immunohistochemical analysis of proliferating and differentiated cells in the fetal brain (diencephalon) of Control and PMH groups at embryonic days e14, e16, e18, and e20. (**A**) Quantification of Ki67-positive cells. Data were analyzed using an ordinary two-way ANOVA with Group (Control vs. PMH) and Time (e14, e16, e18, e20) as factors. The analysis revealed a significant Group × Time interaction (F(3,40) = 9.818, *p* < 0.0001) and a significant main effect of Time (F(3,40) = 9.905, *p* < 0.0001), while the main effect of Group was not significant. Sidak’s multiple comparisons test (Control vs. PMH within each embryonic day) showed a lower number of Ki67-positive cells in the PMH group at e14 (adjusted *p* = 0.0231) and a higher number at e16 (adjusted *p* = 0.0002), with no significant differences at e18 or e20. * adjusted *p* < 0.05, *** adjusted *p* < 0.001 (**B**) Quantification of NeuN-positive cells. Data were analyzed using an ordinary two-way ANOVA with Group (Control vs. PMH) and Time (e14, e16, e18, e20) as factors. The analysis revealed a significant Group × Time interaction (F(3,40) = 3.234, *p* = 0.0322) and a significant main effect of Time (F(3,40) = 3.011, *p* = 0.0413), while the main effect of Group was not significant. Sidak’s multiple comparisons test (Control vs. PMH within each embryonic day) did not detect significant between-group differences at any timepoint. (**C**) Representative immunohistochemical images of Ki67 (violet) and NeuN (green) staining with nuclear counterstain (DAPI, blue) in fetal brain sections. Scale bars (150 μm) as indicated. Bars represent mean ± SEM; dots indicate individual values, n = 6 per group.

The number of proliferating cells (Ki67-positive) and differentiated neurons (NeuN-positive) in the fetal brain (diencephalon) was evaluated by immunohistochemistry on e14, e16, e18, and e20 (Figure 2). Immunohistochemical analysis of Ki67-positive cells revealed a decrease in their number in PMH fetuses at e14 (Figure 2A,C, PMH vs. Control, *p* < 0.05, Sidak’s multiple comparisons test) and a likely compensatory increase at e16 (PMH vs. Control, *p* < 0.001, Sidak’s multiple comparisons test). No significant differences between the PMH and Control groups were detected at e18 or e20 (Figure 2A,C). Immunohistochemical analysis of NeuN-positive cells showed no significant differences between the PMH and Control groups at e14, e16, e18, or e20 (Figure 2B,C).

### 2.2. Consequences of Maternal Hypoxia on the HIF1α Content in the Fetal Part of Placenta and Embryonic Brain in Next Generation

**Figure 3:**
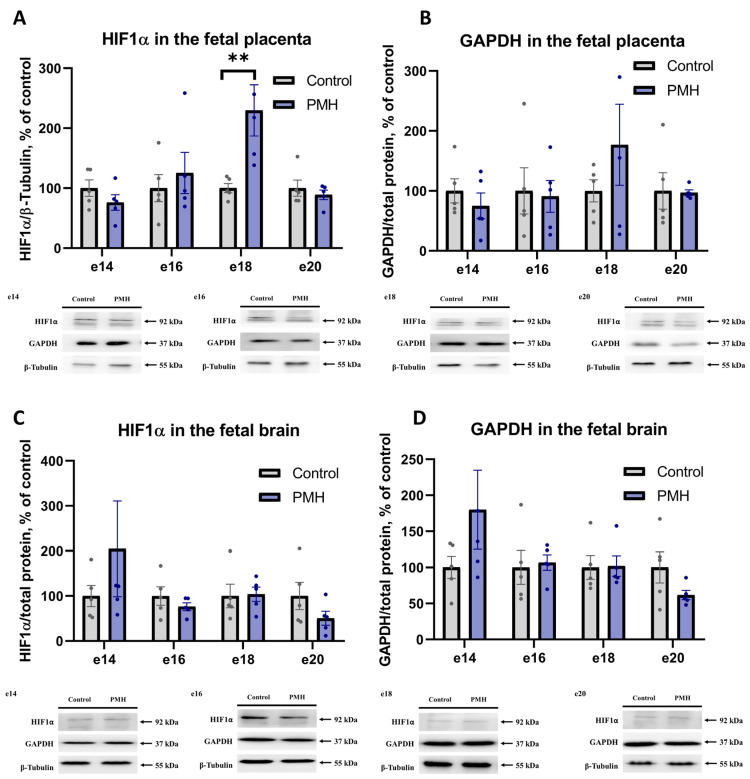
Western blot analysis of HIF1α and GAPDH protein content in the fetal placenta (FP) and embryonic brain of Control and PMH groups at embryonic days e14, e16, e18, and e20. (**A**) Quantification of HIF1α protein content in the fetal placenta. Data were analyzed using an ordinary two-way ANOVA with Group (Control vs. PMH) and Time (e14, e16, e18, e20) as factors. The analysis revealed a significant Group × Time interaction (F(3,32) = 4.638, *p* = 0.0084) and a significant main effect of Time (F(3,32) = 4.638, *p* = 0.0084), while the main effect of Group was not significant. Sidak’s multiple comparisons test (Control vs. PMH within each embryonic day) showed significantly higher placental HIF1α protein content in the PMH group at e18 (adjusted *p* = 0.0014), with no significant differences at e14, e16, or e20. ** adjusted *p* < 0.01. Representative immunoblots are shown below the graph. (**B**) Quantification of GAPDH protein content in the fetal placenta. No significant differences between the Control and PMH groups were detected at any examined developmental stage. (**C**) Quantification of HIF1α protein content in the embryonic brain. No significant differences between the Control and PMH groups were detected at any examined developmental stage. Representative immunoblots are shown below the graph. (**D**) Quantification of GAPDH protein content in the embryonic brain. No significant differences between the Control and PMH groups were detected at any examined developmental stage. Representative immunoblots are shown below the graph. Bars represent mean ± SEM; dots indicate individual values, n = 5 per group.

HIF1α protein content and the HIF1-dependent protein GAPDH were analyzed in the fetal placenta (FP) and fetal brain on embryonic days e14, e16, e18, and e20 by Western blotting (Figure 3). In the FP, HIF1α protein content was increased at e18 in the PMH group (Figure 3A, PMH vs. Control, *p* < 0.01, Sidak’s multiple comparisons test), and no significant group differences were detected in the GAPDH protein content at any of the studied developmental stages (Figure 3B). In the embryonic brain, no significant group differences were detected in either HIF1α or GAPDH protein content at any of the studied developmental stages (Figure 3C,D).

### 2.3. Transgenerational Effect of Maternal Hypoxia on the Neurons and Astrocytes Content in the Adult Brain Structures

**Figure 4:**
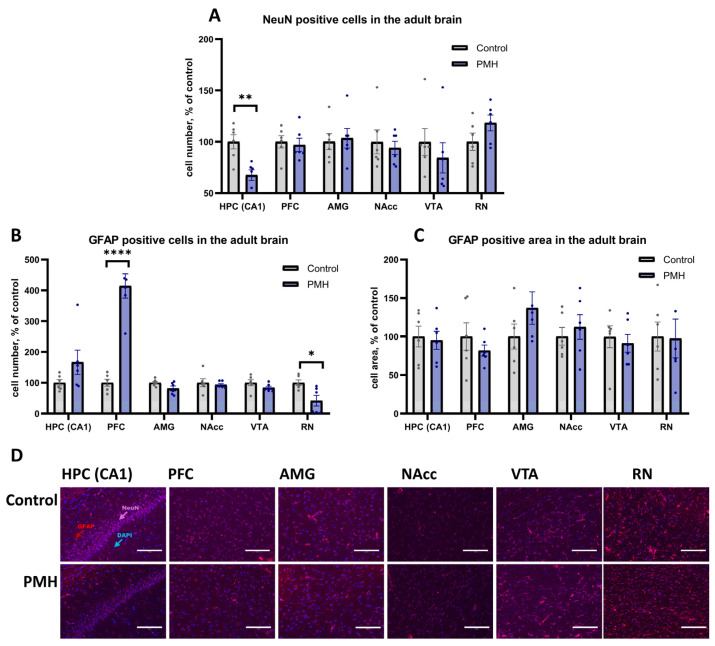
Immunohistochemical analysis of neuronal and astrocytic markers in adult brain structures of Control and PMH groups. (**A**) Quantification of NeuN-positive cells in the HPC, PFC, AMG, NAcc, VTA, and RN. PMH rats showed a reduced number of NeuN-positive cells in the HPC (CA1) (unpaired Student’s *t*-test, *p* = 0.0037), whereas no significant differences were detected in the other examined brain regions. ** adjusted *p* < 0.01 (**B**) Quantification of GFAP-positive cells in the HPC, PFC, AMG, NAcc, VTA, and RN. PMH rats showed an increased number of GFAP-positive cells in the PFC (unpaired Student’s *t*-test, *p* < 0.0001) and a decreased number in the RN (Mann–Whitney U test, *p* = 0.0152), while no significant differences were observed in the remaining structures. * adjusted *p* < 0.05, **** adjusted *p* < 0.0001 (**C**) Quantification of GFAP-positive area in the HPC, PFC, AMG, NAcc, VTA, and RN. No significant differences between the Control and PMH groups were detected in any analyzed brain region. (**D**) Representative immunohistochemical images of NeuN (violet) and GFAP (red) staining with nuclear counterstain (DAPI, blue) in adult brain sections. Scale bars (125 μm) as indicated. Bars represent mean ± SEM; dots indicate individual values, n = 6 per group.

The number of neurons (NeuN-positive cells), as well as the number and area of astrocytes (GFAP-positive cells and GFAP-positive area), were assessed in the hippocampus (HPC, CA1), prefrontal cortex (PFC), amygdala (AMG), nucleus accumbens (NAcc), ventral tegmental area (VTA), and raphe nuclei (RN) of adult rats by immunohistochemistry (Figure 4). Analysis of NeuN-positive cells revealed a decrease in neuronal number in the HPC (CA1) of PMH rats (Figure 4A,D, PMH vs. Control, *p* = 0.0037, unpaired Student’s *t*-test), with no significant changes in the other examined brain regions (Figure 4A,D). Analysis of GFAP-positive cells demonstrated an increased astrocyte number in the PFC (Figure 4B,D, PMH vs. Control, *p* < 0.0001, unpaired Student’s *t*-test) and a decreased astrocyte number in the RN (Figure 4B,D, PMH vs. Control, *p* = 0.0152, Mann–Whitney U test) of PMH rats, whereas no significant differences were detected in the remaining structures. No significant differences in GFAP-positive area were found in any of the analyzed brain regions (Figure 4C,D).

### 2.4. Transgenerational Effect of Maternal Hypoxia on the HIF1a and HIF1-Dependent mRNA and Protein Expression in the Adult Brain Structures

**Figure 5:**
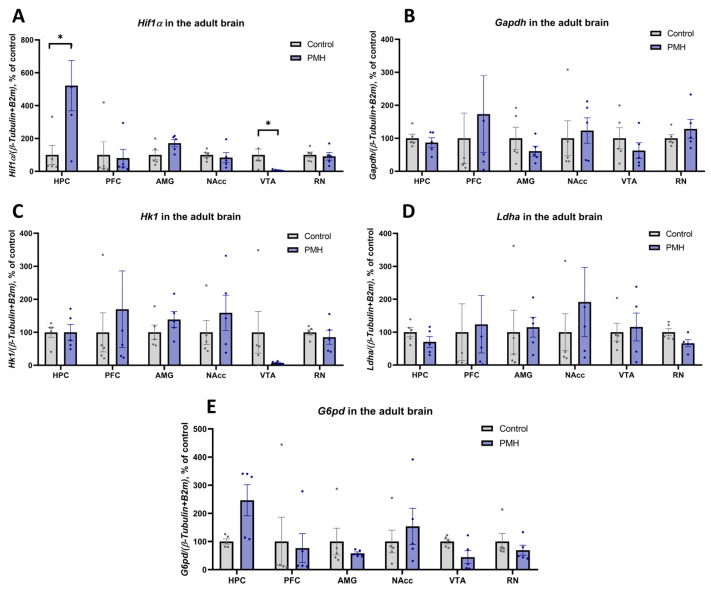
qRT-PCR analysis of *Hif1α* and HIF1-dependent mRNA expression in adult brain structures of Control and PMH groups. (**A**) Relative *Hif1α* mRNA expression in the HPC, PFC, AMG, NAcc, VTA, and RN. PMH rats showed increased Hif1α mRNA expression in the HPC (Mann–Whitney U test, *p* = 0.0317) and decreased *Hif1α* mRNA expression in the VTA (unpaired Student’s *t*-test, *p* = 0.0336), while no significant differences were detected in the other analyzed structures. * adjusted *p* < 0.05 (**B**) Relative *Gapdh* mRNA expression in the HPC, PFC, AMG, NAcc, VTA, and RN. No significant differences between the Control and PMH groups were detected in any analyzed brain region. (**C**) Relative *Hk1* mRNA expression in the HPC, PFC, AMG, NAcc, VTA, and RN. No significant differences between the Control and PMH groups were detected in any analyzed brain region. (**D**) Relative *Ldha* mRNA expression in the HPC, PFC, AMG, NAcc, VTA, and RN. No significant differences between the Control and PMH groups were detected in any analyzed brain region. (**E**) Relative *G6pd* mRNA expression in the HPC, PFC, AMG, NAcc, VTA, and RN. No significant differences between the Control and PMH groups were detected in any analyzed brain region. Bars represent mean ± SEM; dots indicate individual values, n = 5 per group.

**Figure 6:**
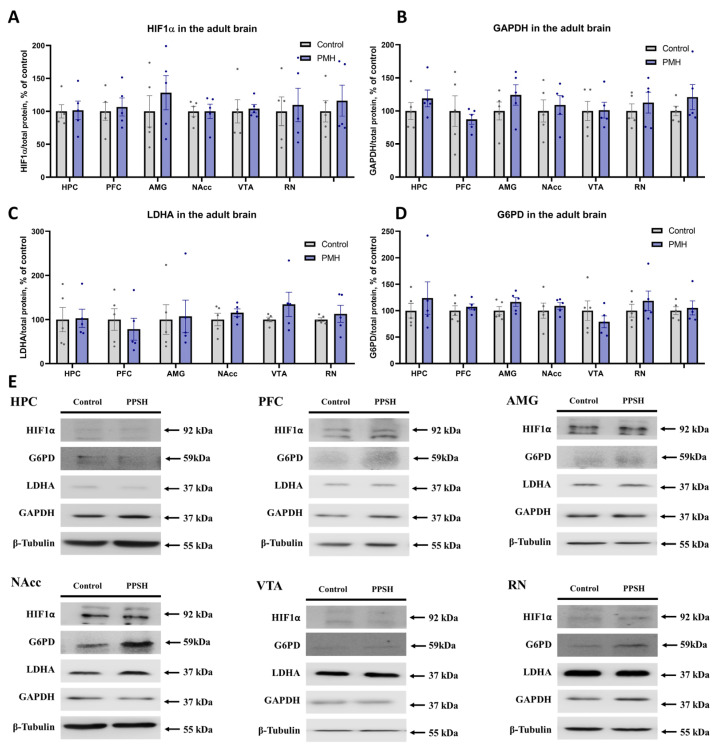
Western blot analysis of HIF1-related protein content in adult brain structures of Control and PMH groups. (**A**) Quantification of HIF1α protein content in the HPC, PFC, AMG, NAcc, VTA, and RN. No significant differences between the Control and PMH groups were detected in any analyzed brain region. (**B**) Quantification of GAPDH protein content in the HPC, PFC, AMG, NAcc, VTA, and RN. No significant differences between the Control and PMH groups were detected in any analyzed brain region. (**C**) Quantification of LDHA protein content in the HPC, PFC, AMG, NAcc, VTA, and RN. No significant differences between the Control and PMH groups were detected in any analyzed brain region. (**D**) Quantification of G6PD protein content in the HPC, PFC, AMG, NAcc, VTA, and RN. No significant differences between the Control and PMH groups were detected in any analyzed brain region. (**E**) Representative immunoblots for the analyzed proteins in the corresponding brain structures. Bars represent mean ± SEM; dots indicate individual values, n = 5 per group.

HIF1α mRNA and protein levels, as well as HIF1-dependent transcriptional products, were evaluated in the HPC, PFC, AMG, NAcc, VTA, and RN of adult rats using quantitative RT PCR and Western blotting (Figure 5 and Figure 6). Relative *Hif1α* mRNA expression was increased in the HPC (Figure 5A, PMH vs. Control, *p* = 0.0317, Mann–Whitney U test) and decreased in the VTA (Figure 5A, PMH vs. Control, *p* = 0.0336, unpaired Student’s *t*-test) PMH rats. However, no significant changes in HIF1α protein content were detected in any of the examined brain structures (Figure 6A,E). Among HIF1-dependent targets, PMH rats showed no significant differences in relative *Gapdh*, *Hk1*, *Ldha* and *G6pd* mRNA expression (Figure 5B–E) or in GAPDH, LDHA and G6PD protein levels (Figure 6B–E) in any of the examined brain structures.

## 3. Discussion

The present study demonstrates that maternal hypoxia induces transgenerational developmental programming effects in the next generation, manifested at both fetal and adult stages. The main findings include reduced fetal brain weight at e16–e18, a stage-dependent alteration in proliferating activity (Ki67), increased HIF1α protein content in the fetal placenta at e18 without a parallel increase in the fetal brain, and region-specific structural changes in adult offspring, including reduced neuronal number in the hippocampus, increased astrocyte number in the prefrontal cortex, and decreased astrocyte number in the raphe nuclei. In contrast, adult PMH offspring showed only modest and region-specific changes in *Hif1*α and selected HIF1-related transcripts, without corresponding protein-level changes.

One of the key observations is the stage-dependent change in Ki67-positive cell counts (reduced at e14 and increased at e16), suggesting altered proliferative dynamics during early neurodevelopment and persistent reduction in fetal brain weight from e16 onward. Although an important limitation of this study is that we did not control for fetal sex, this pattern suggests that maternal hypoxia in the previous generation may alter the timing and dynamics of neurodevelopment in the next generation, rather than causing a stable suppression of proliferation or differentiation throughout gestation. Such a pattern is compatible with a developmental delay or shift in neurogenic maturation, with partial compensation at later stages. However, this apparent cellular normalization is insufficient to restore normal brain growth, as reflected by the sustained reduction in fetal brain weight. This interpretation fits well with the concept of developmental programming, where early perturbations of developmental timing can have long-term structural consequences even in the absence of persistent abnormalities in the same markers at later stages [4,18,19].

A particularly important result is the increase in HIF1α protein content in the fetal placenta (e18) in the PMH group, while no corresponding increase was detected in the fetal brain. This finding supports the idea that the placenta is a more sensitive or earlier-responding compartment in the transgenerational response to maternal hypoxia. The placenta should be viewed not as a passive conduit but as an active mediator of developmental programming [19,20,21,22,23,24,25]. A placental hypoxia-related response may influence fetal neurodevelopment through multiple mechanisms, including altered nutrient and oxygen delivery, changes in glucocorticoid exposure, inflammatory signaling, and growth factor regulation [20,21,22,23,24,25]. Thus, even in the absence of detectable HIF1α elevation in fetal brain tissue, placental adaptation may be sufficient to modify fetal brain developmental trajectories [19,20,21,22,25].

A major limitation is that fetal samples were collected without sex determination, and therefore fetal outcomes represent a composite of male and female fetuses. Given the extensive evidence for sexually dimorphic responses to gestational stressors, sex-specific effects could have diluted group differences or increased variance, particularly for cellular and molecular endpoints. In addition, adult analyses were performed in males; therefore, extrapolation to females requires direct testing. Future studies will incorporate sex as a mandatory biological variable across developmental stages.

The adult PMH phenotype is characterized by selective regional alterations rather than generalized brain-wide changes. The most prominent neuronal effect was a reduction in NeuN-positive cell number in the hippocampus (CA1), a structure critically involved in memory, stress integration, and emotional regulation. This finding is consistent with the known developmental vulnerability of the hippocampus to prenatal stress and hypoxic insults and supports the idea that maternal hypoxia can produce long-term transgenerational effects on limbic system organization [4,6]. The astroglial findings further strengthen this interpretation. PMH offspring showed an increased number of GFAP-positive astrocytes in the PFC and a decreased number in the raphe nuclei, with no significant changes in GFAP-positive area. This pattern suggests region-specific astrocytic remodeling. Increased astrocyte number in the PFC may reflect long-term compensatory or reactive changes. In contrast, reduced astrocytic representation in the raphe nuclei may indicate impaired trophic or metabolic support in a monoaminergic regulatory center [26,27]. Although neurotransmitter-specific markers were not analyzed in the present study, the raphe finding is particularly relevant because this region is central to serotonergic regulation of affective behavior and stress responsivity [28]. Together with hippocampal and prefrontal changes, the results suggest that maternal hypoxia may transgenerationally reprogram neuroglial architecture in circuits involved in cognition and emotional regulation [4,6,26,27,28].

In the adult brain, the observed molecular changes in HIF1-related markers were limited, region-specific, and not consistently reflected at the protein level. PMH offspring showed increased *Hif1α* mRNA in the hippocampus and decreased *Hif1α* mRNA in the VTA. However, no significant differences were detected in HIF1α, GAPDH, LDHA, or G6PD protein levels in the analyzed structures. This transcript–protein dissociation suggests that the long-term transgenerational effects of maternal hypoxia are not maintained as constitutive activation of HIF1-dependent metabolic pathways in adulthood. Several explanations are possible. First, HIF1 signaling is strongly regulated at post-transcriptional and post-translational levels, and mRNA abundance may not reliably predict steady-state protein content [29,30]. Second, region-level homogenates may mask cell-type-specific changes, particularly in heterogeneous structures [30,31]. Third, programming effects may be expressed as altered pathway reactivity under challenge rather than baseline differences under resting conditions [26,28]. Accordingly, the present data support a model in which HIF1 signaling may play a role during placental and/or early developmental stages, while the persistent adult phenotype is expressed primarily through structural and cellular remodeling [20,25,29].

The combined fetal, placental, and adult findings fit a multilevel developmental programming model. In this model, maternal hypoxia in the previous generation induces persistent changes in maternal physiology and placental function, which then alter the intrauterine environment of the next generation. This altered environment affects fetal brain developmental timing and growth, and these early changes are later manifested as region-specific neuronal and astrocytic remodeling in adulthood [19,21,22,23,24,25,32,33]. This interpretation is also consistent with the broader literature showing that prenatal stress/hypoxia-related exposures can induce long-lasting changes in epigenetic regulation, placental signaling, and neurodevelopmental trajectories across generations [19,22,32,33]. The present study extends this framework by showing that, in the next generation, the most robust effects are structural and developmental, while baseline molecular changes in HIF1-dependent metabolic markers are comparatively subtle. Thus, HIF1 should be considered a component of the programming network but not the sole determinant of the adult transgenerational phenotype [20,25,29]. Maternal hypoxia and related perturbations of maternal–fetal oxygenation are clinically relevant in a range of pregnancy complications, including placental insufficiency, hypertensive disorders of pregnancy (e.g., preeclampsia), maternal respiratory compromise, and high-altitude pregnancy. In these settings, placental adaptive responses can shape fetal neurodevelopment via changes in oxygen/nutrient delivery, endocrine signaling, and inflammatory milieu. Within this framework, our observation of an e18-specific increase in placental HIF1α protein content, together with persistent, region-specific neuroglial alterations in adult offspring, supports the concept that placental signaling may act as a mediator of long-term developmental programming, including across generations. Importantly, because the offspring analyzed here were not directly exposed to hypoxia, the findings are most consistent with a programming mechanism rather than direct hypoxic injury.

## 4. Materials and Methods

### 4.1. Animals

The study was carried out using animals from the CCU “Biocollection of laboratory mammals of different taxonomic affiliation” of the Pavlov Institute of Physiology of RAS. Adult pregnant female Wistar rats and their first- and second-generation offspring were utilized. All experimental procedures were performed in compliance with the Guidelines for Reporting Animal Research [34] and were approved by the Ethical Committee for the Use of Animal Subjects at the Pavlov Institute of Physiology (protocol no. 09/15 of 15 September 2025). The animals were maintained under standard laboratory conditions, with unrestricted access to food and water, a 12:12 light/dark cycle, ambient room temperature, and constant humidity of approximately 60%. Rat pups were weaned at 30 days of age, a time when dams spent no more than 2 h nursing [35]. This postpartum day aligns with our prior studies on prenatal pathologies, mitigating the stress typically associated with weaning. After weaning, the rats were housed in cages 60 cm × 30 cm × 20 cm in size, with 5–6 animals in each. During all the experiments, the researchers were blinded to the allocation of the groups.

### 4.2. Maternal Hypoxia and Progeny of Maternal Hypoxia

**Figure 7:**
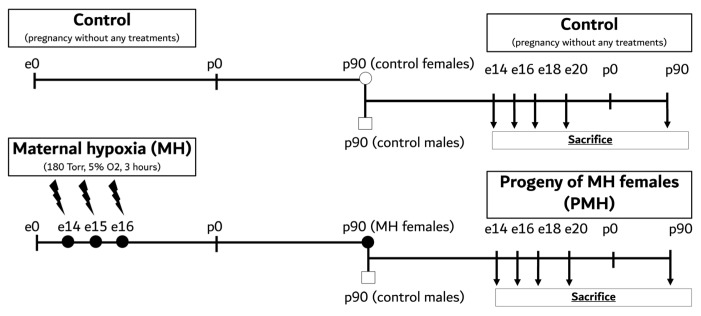
Schematic outline of the experimental study design. MH, maternal hypoxia; PMH, progeny of maternal hypoxia; e0–20, embryonic days; p0, p90, postnatal days.

Maternal hypoxia (MH) reported in our previous studies was used as a reliable model combining the fetal hypoxia and maternal stress response during pregnancy [6,9,15,16,17]. To model MH, we used a flow-type hypobaric chamber at a temperature of 20 °C to 25 °C in which atmospheric pressure was gradually reduced to 180 Torr, reaching 5% of oxygen content (equivalent to 11,000 m above sea level) during 20 min. The experimental chamber was partitioned into six distinct compartments, thereby facilitating the simultaneous modeling of MH on six pregnant rats. After 3 h of treatment the oxygen content was returned to normal within 20 min. Pregnant dams were treated under such conditions for 3 consecutive days (e14–e16) with an interval of 24 h between the sessions (Figure 7). The mortality rate in the hypobaric chamber was around 15%. Intact control females were also placed in the hypobaric chamber for 3 h on the 14th, 15th, and 16th days of pregnancy without being subjected to hypoxic or hypobaric exposure. In further experiments, we used adult male offspring with active spermatogenesis from the Control group and adult females from the Control and MH groups at the age of 3 months. Control males were added to Control and MH females, after which the progeny of the Control and maternal hypoxic females (PMH) were reared. In further experiments, we used embryonic (e14, e16, e18, e20) offspring without sex definition and adult male offspring from the Control and PMH groups at the age of 3 months (Figure 7). A total of 120 Wistar rats were used in this study: 44 control and 44 PMH fetuses were obtained from 6 control and 6 MH pregnant females; 16 control and 16 PMH adult males were grown from 6 control and 6 PMH litters (born by different females). Each rat group consisted of randomly selected rats from different dams to minimize litter bias without any inclusion or exclusion criteria in the experiment. Sample sizes: immunohistochemistry n = 6 per group, biochemical assays n = 5 per group.

### 4.3. Sample Preparation

To collect the samples for further analysis, tissues from the embryonic brains and fetal placenta (FP) (e14, e16, e18, e20) of control and PMH rats were dissected. Total embryonic brain weight was measured (information on the level of brain water content was not taken into account). Embryonic brain and FP samples were frozen in liquid nitrogen for subsequent total protein extraction and Western blotting. For immunohistochemistry, brain samples were fixed in FineFix (Milestone, Sorisole, Italy).

Adult control and PMH rats were sacrificed by guillotine at the age of 3 months. Immediately after decapitation, the brain was removed and sectioned in the frontal plane into three parts (along the optic chiasm and after the mammillary nuclei of the hypothalamus). Brain structure dissection was performed on a cooling table under digital microscope guidance. The average procedure time from decapitation to dissection of all necessary brain structures was 1.5 to 2 min. Hippocampus (HPC), medial prefrontal cortex (PFC), amygdala (AMG), nucleus accumbens (NAcc), ventral tegmental area (VTA), and dorsal raphe nuclei (RN) samples for mRNA analysis were quickly extracted from the brain and homogenized in ExtractRNA reagent (ExtractRNA Kit, BC032, Evrogen, Moscow, Russia). HPC, PFC, AMG, NAcc, VTA, RN samples were dissected and frozen in liquid nitrogen for subsequent total protein extraction and Western blotting. For immunohistochemistry, brain samples containing HPC, PFC, AMG, NAcc, VTA, RN were fixed in FineFix (Millestone, Italy).

### 4.4. Immunohistochemistry

For immunohistochemistry samples of embryonic brains and adult brain samples containing HPC, PFC, AMG, NAcc, VTA, RN were submerged in a fixative solution (28 mL FineFix (Milestone, Italy) + 72 mL 96% ethanol) for 24 h following a standard histological protocol. Dehydration of the tissues was achieved by treatment with 70% ethanol for 1.5 h, 96% ethanol: 100% isopropyl alcohol (80:20) for 3 h and 100% isopropyl alcohol for 3 h at 60 °C. The samples were then immersed in liquid paraffin (twice, for 1 h) at 56 °C and sectioned. The 7 µm sagittal sections of the embryonic brain and 7 µm coronal sections of the adult brain were prepared using a rotary microtome (Sakura Accu-Cut, Tokyo, Japan). Sections were mounted onto poly-L-lysine-covered slides, deparaffinized in xylol (twice, for 5 min), and rehydrated in alcohols (96% → 96% → 96% → 70% for 5 min in each solution).

Embryonic central sagittal sections containing diencephalon were incubated overnight at 4 °C with mouse primary antibodies against NeuN (1:400, ab104224, Abcam, Cambridge, UK) and rabbit primary antibodies against Ki67 (1:100, ab16667, Abcam, UK). Brain sections containing HPC (−3 mm from Bregma), PFC (+2.5 mm from Bregma), AMG (−3 mm from Bregma), NAcc (+2.52 mm from Bregma), VTA (−5 mm from Bregma), and RN (−7.5 mm from Bregma) were incubated overnight at 4 °C with mouse primary antibodies against NeuN (1:400, ab104224, Abcam, UK) and rat primary antibodies against GFAP (1:1000, 2.2B10, Invitrogen, Carlsbad, CA, USA). After incubation with the primary antibodies and washing 3 times in PBS, all sections were incubated with secondary goat anti-rabbit AF647 (1:200, E-AB-1075, Elabscience Biotechnology, Houston, TX, USA), anti-mouse AF488 (1:200, E-AB-1056, Elabscience Biotechnology, USA) and donkey anti-rat AF594 (1:200, A-21209, Invitrogen, USA) antibodies for 30 min and then washed three times. Afterwards, the sections were then incubated with DAPI reagent for 15 min (E-IR-R103, Elabscience Biotechnology, USA). The sections were washed, dried, covered with permanent mountant (Sub-X Mounting Medium; Leica Biosystems, Deer Park, IL, USA), and analyzed using an EVOS M5000 fluorescence microscope (Invitrogen, USA). The number of NeuN and Ki67 positive cells (for embryonic diencephalon), the number of NeuN positive cells and GFAP positive cells, and the area were quantified in a field of 200 μm × 200 μm using ImageJ 1.54 software. The data obtained from four slices per brain (with one field per slice analyzed) were averaged.

### 4.5. Western Blotting

Protein expression in the FP, embryonic brains and adult rat brain structures was assessed by Western blotting. Samples were homogenized in ice-cold 50 mM Tris-HCl buffer (TBS, pH 8.0) containing 150 mM NaCl, 1% Triton X-100, and a protease/phosphatase inhibitor cocktail (SB-G2006, SB-G2007; Servicebio, Wuhan, China). Homogenates were incubated on a shaker for 30 min at +4 °C, followed by centrifugation at 14,000× *g* for 10 min. The resulting supernatants were collected, and equal amounts of total protein were mixed with 3× Laemmli buffer and heated at +70 °C for 10 min. Proteins were separated by sodium dodecyl sulfate–polyacrylamide gel electrophoresis (10% resolving gel was used for all proteins) and transferred onto PVDF membranes (Thermo Fisher Scientific, Waltham, MA, USA). Membranes were blocked for 1 h in PBS containing 5% skimmed milk and incubated for 2 h at room temperature with rabbit primary antibodies against HIF1α (1:2000, AF1009, Affinity Biosciences, Cincinnati, OH, USA), LDHA (1:5000, AF7672, Affinity Biosciences, USA), G6PD (1:2000, DF6444, Affinity Biosciences, USA), GAPDH (1:5000, AF7021, Affinity Biosciences, USA), or β-Tubulin (1:5000, AF7011, Affinity Biosciences, USA). Following three washes in PBST (PBS with 0.1% Tween 20), membranes were incubated for 1 h at room temperature with HRP-conjugated anti-rabbit secondary antibodies (1:5000, E-AB-1003; Elabscience Biotechnology, USA) and subsequently washed twice with PBST. Immunoreactive bands were visualized using the Clarity ECL chemiluminescence detection kit (Bio-Rad, Hercules, CA, USA) and imaged with the ChemiScope 6000 system (Clinx Science Instruments, Shanghai, China). Densitometric analysis was performed using ImageJ 1.54 software (NIH), and protein levels were normalized to total protein detected by amido black staining. β-Tubulin is presented as a loading control. Full Western blots are presented in the Supplementary Materials.

### 4.6. Quantitative RT PCR

Total RNA from the embryonic brains and adult rat brain structures was extracted using the ExtractRNA Kit (BC032, Evrogen, Russia) and purified with the CleanRNA Standard Kit (BC033, Evrogen, Russia) according to the manufacturer’s instructions. The quality and concentration of total RNA were determined by measuring optical density at 260 nm and 280 nm using a spectrophotometric microplate reader (CLARIOstar PLUS, BMG Labtech, Ortenberg, Germany). Complementary DNA (cDNA) was synthesized from 2 μg of total RNA using the MMLV Reverse Transcription Kit (SK021, Evrogen, Russia). Quantitative real-time PCR was performed using qPCRmix-HS SYBR+LowROX (PK156L, Evrogen, Russia) on a Locus Intero 6 Thermal Cycler (Tianlong, Xi’an, China). Primer sequences, annealing temperatures, and amplicon sizes are listed in Table 1. Target gene expression levels were calculated using the ∆∆Ct method, with *β-Tubulin* and *B2m* mRNA serving as the reference genes for normalization. Reference genes were chosen from a panel of candidate housekeeping genes using the BestKeeper algorithm.

### 4.7. Statistical Analysis

Statistical analyses were performed in GraphPad Prism 8 (GraphPad Software, La Jolla, CA, USA). For each comparison, group distributions were assessed with the Shapiro–Wilk test (α = 0.05) and by inspection of Q–Q plots. Normally distributed data were analyzed with parametric tests: unpaired two-tailed Student’s *t*-test for two-group comparisons and ordinary two-way ANOVA with group and time as factors. For multiple comparisons after analysis, the Sidak test was used. When normality was not met, the Mann–Whitney U test was applied. Data are presented as mean ± SEM. Statistical significance was set at *p* < 0.05 (two-tailed). Exact n and the test used are provided in the figure legends.

## 5. Conclusions

In conclusion, the present study shows that maternal hypoxia induces transgenerational effects in the next generation that are expressed as altered fetal brain development and region-specific neuroglial remodeling in adulthood. These findings support the concept that the long-term consequences of maternal hypoxia are mediated primarily by developmental programming mechanisms involving placental adaptation, endocrine regulation, and likely epigenetic transmission, rather than by sustained global HIF1 activation in adult brain tissue [20,21,22,23,24,25,29,32,33]. Future studies integrating behavioral testing and cell-type-resolved analyses will be important for defining the precise mechanisms linking placental hypoxia-related responses to adult brain dysfunction in transgenerational models. A fundamental limitation of the presented findings is the lack of consideration of sex-specific characteristics, which should be given significant attention [36,37,38], particularly in fetal data where sex differentiation was not performed. This issue also requires further research.

## Figures and Tables

**Table 1:** Primers used for quantitative RT PCR.

Gene	Primer Sequences (5′–3′)	Annealing T (°C)	Product Size (bp)
*β-Tubulin*	Forward TAGAGGAGATGCTACTTA Reverse AATGGTGATAATACTGTTAA	58	147
*B2m*	Forward TTAGCAGCCTAGCAGTTC Reverse ACCACTTCACTTCACTCTG	58	134
*Gapdh*	Forward CATTCTTCCACCTTTGAT Reverse CTGTAGCCATATTCATTGT	57	92
*G6pd*	Forward AAGATGATGACCAAGAAG Reverse TTGTATCTGTTGCCATAG	56	80
*Hk1*	Forward CTGGACTGTGGAATCTTG Reverse AGTAAGGAGGCTACATCAT	56	80
*Hif1α*	Forward CCATTCCTCATCCATCAA Reverse CCATCAACTCAGTAATCCT	56	114
*Ldha*	Forward CGAGAGCATAATGAAGAAC Reverse TCCTTGATTCCATAGAGAC	56	75

## Data Availability

The original contributions presented in this study are included in the article/Supplementary Materials. Further inquiries can be directed to the corresponding author.

## Supplementary Materials

The following supporting information can be downloaded at: https://www.mdpi.com/article/10.3390/ijms27083421/s1.

## Abbreviations

AMG	Amygdala
B2M	Beta-2 microglobulin
GAPDH	Glyceraldehyde 3-phosphate dehydrogenase
GFAP	Glial fibrillary acidic protein
G6PD	Glucose-6-phosphate dehydrogenase
HK1	Hexokinase 1
HIF1α	Hypoxia-inducible factor 1-alpha
HPC	Hippocampus
Ki67	Marker of proliferation Kiel 67
LDHA	Lactate dehydrogenase A
MH	maternal hypoxia
NAcc	Nucleus accumbens
NeuN	Neuronal nuclei protein
PFC	Prefrontal cortex (medial)
PMH	Progeny of maternal hypoxia
RN	Raphe nuclei (dorsal)
VTA	Ventral tegmental area
