# Supplementary material for: Morphological Features and HIF1-Dependent Processes in the Brain of Progeny of Female Rats Exposed to Maternal Hypoxia
Source: Int J Mol Sci. 2026 Apr 10;27(8):3421. doi: 10.3390/ijms27083421 (PMC13116916; doi:10.3390/ijms27083421)

E14
FP

HIF1α

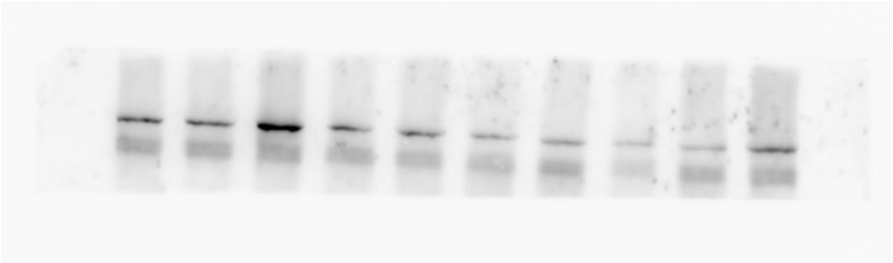

GAPDH

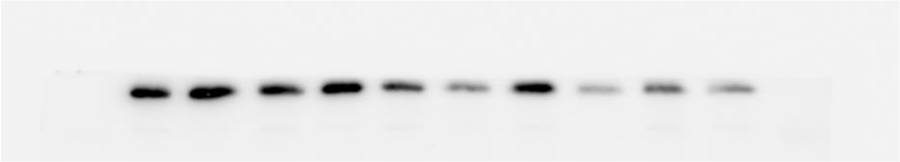

β-Tubulin

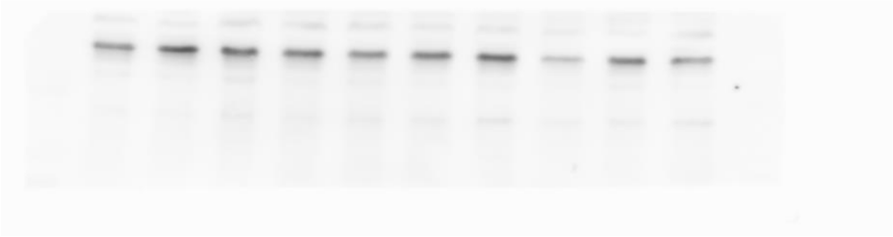

Total protein (AmidoBlack)

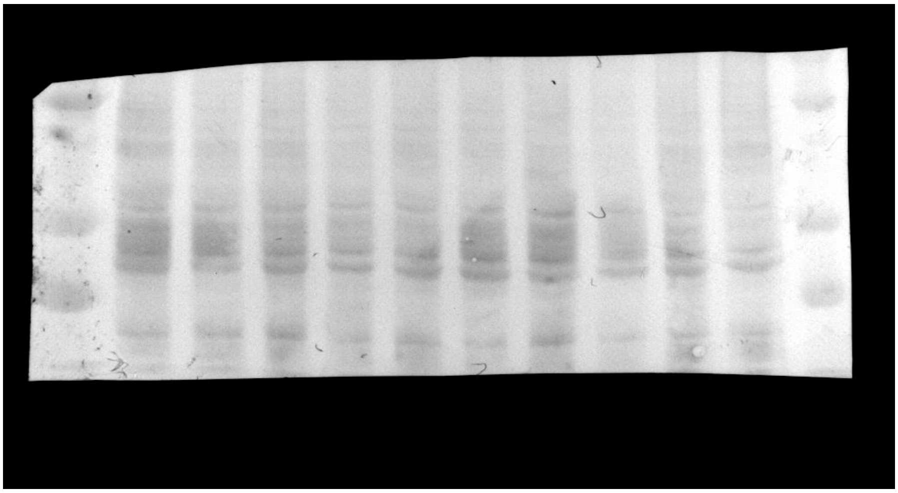

E16
FP

HIF1α

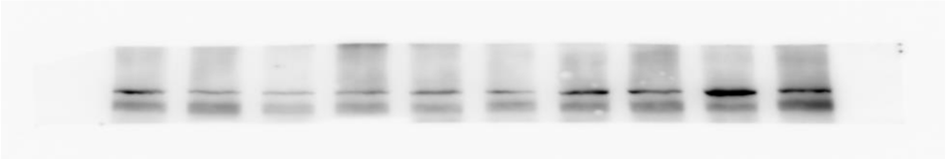

GAPDH

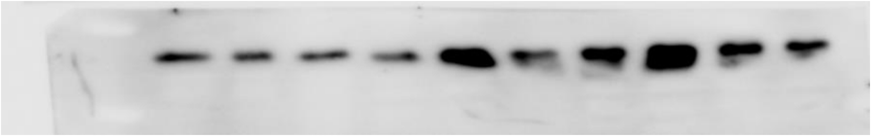

β-Tubulin

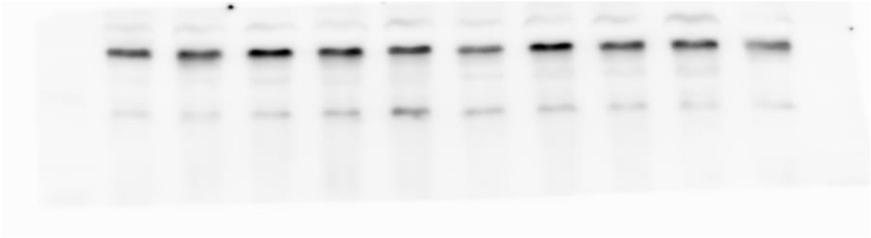

Total protein (AmidoBlack)

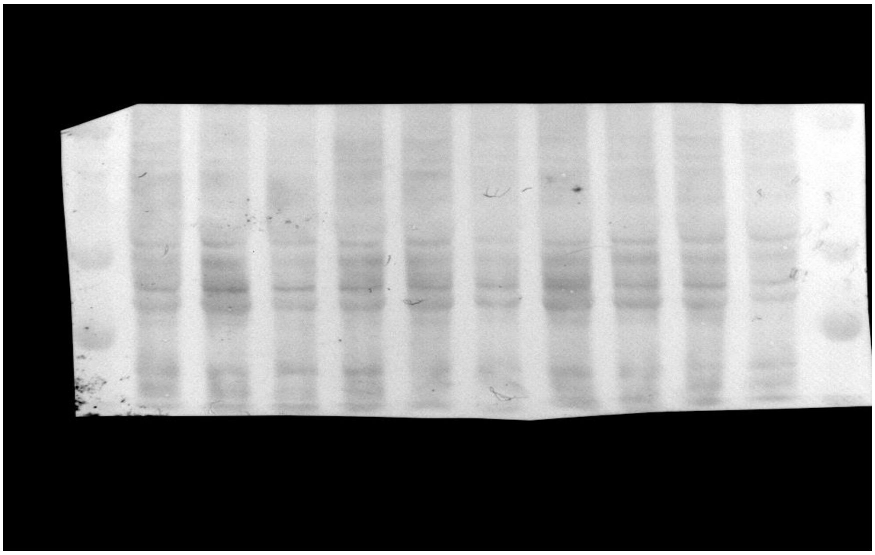

E18
FP

HIF1α

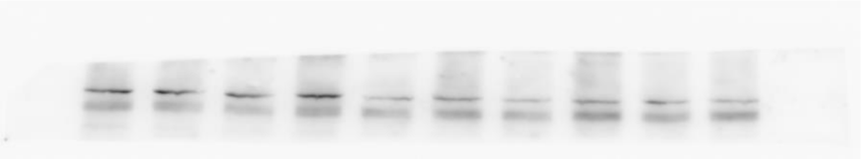

GAPDH

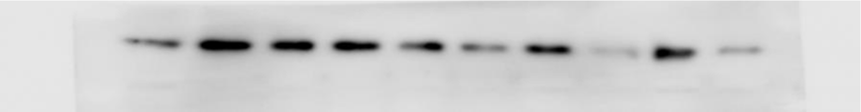

β-Tubulin

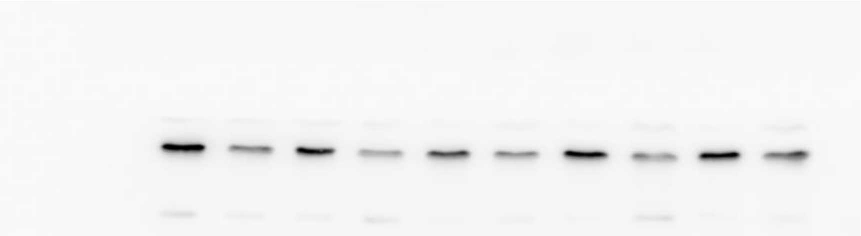

Total protein (AmidoBlack)

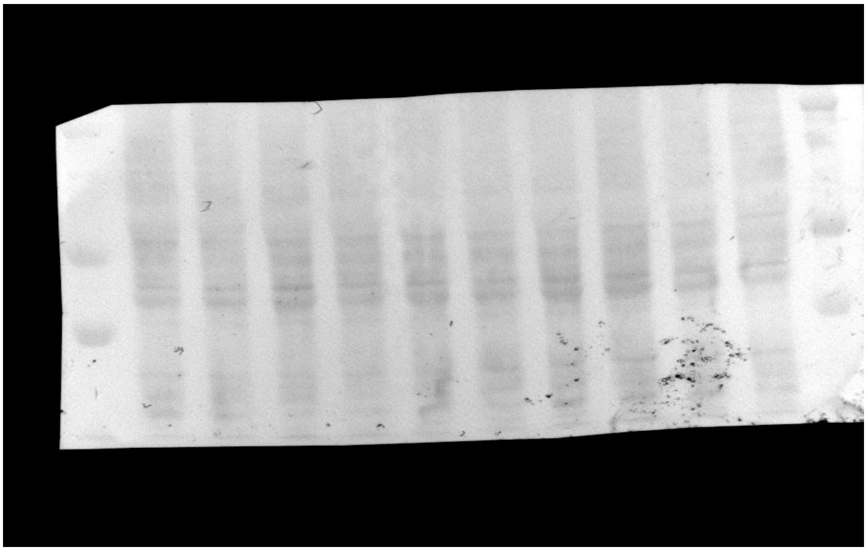

E20
FP

HIF1α

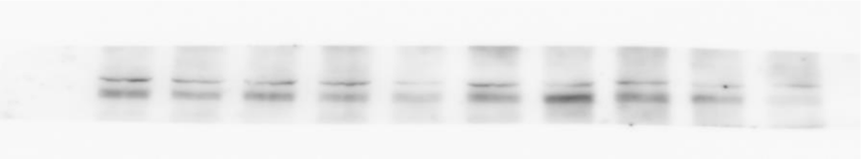

GAPDH

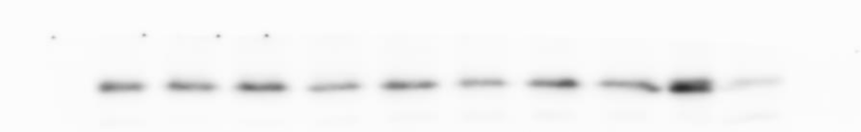

β-Tubulin

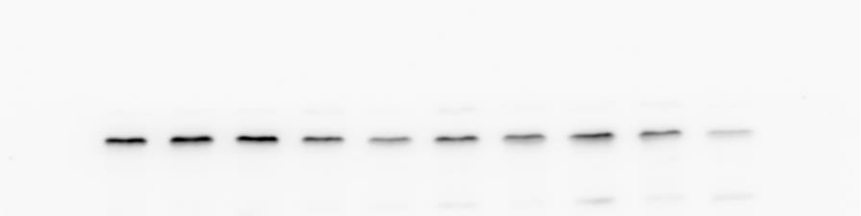

Total protein (AmidoBlack)

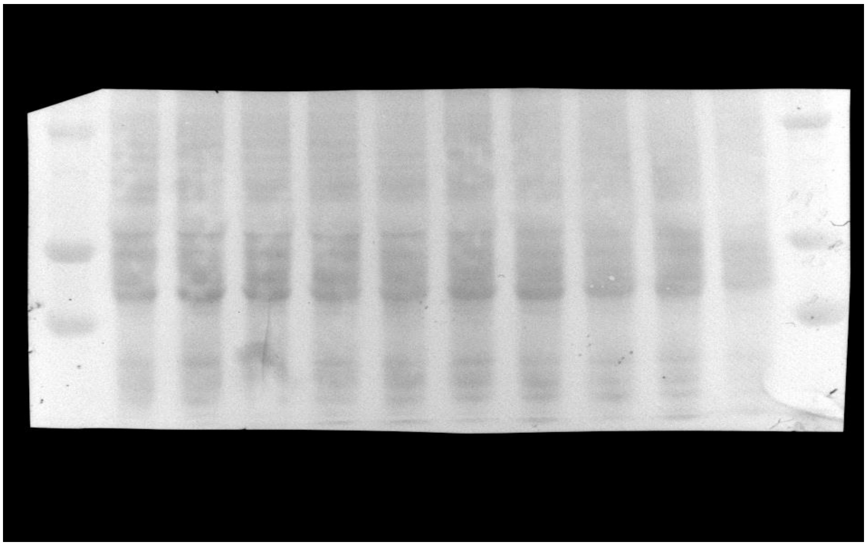

E14
Brain

HIF1α

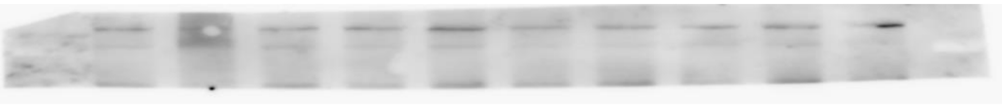

GAPDH

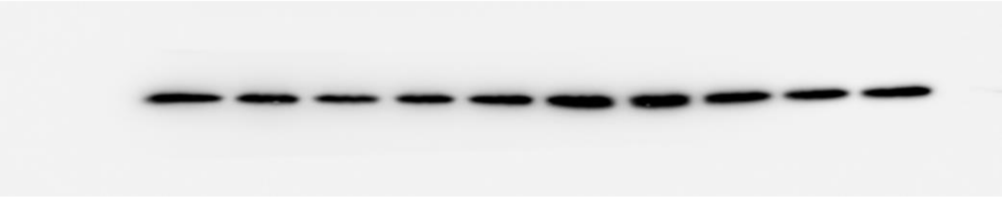

β-Tubulin

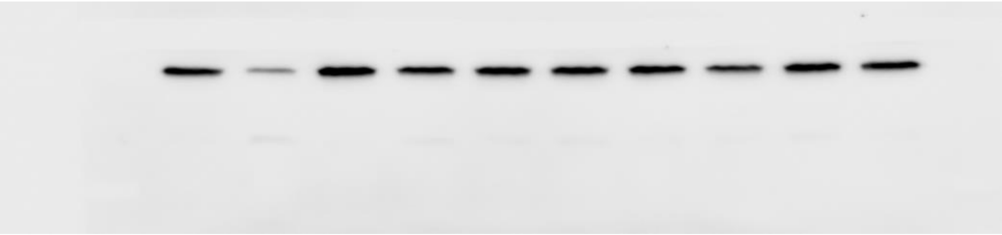

Total protein
(AmidoBlack)

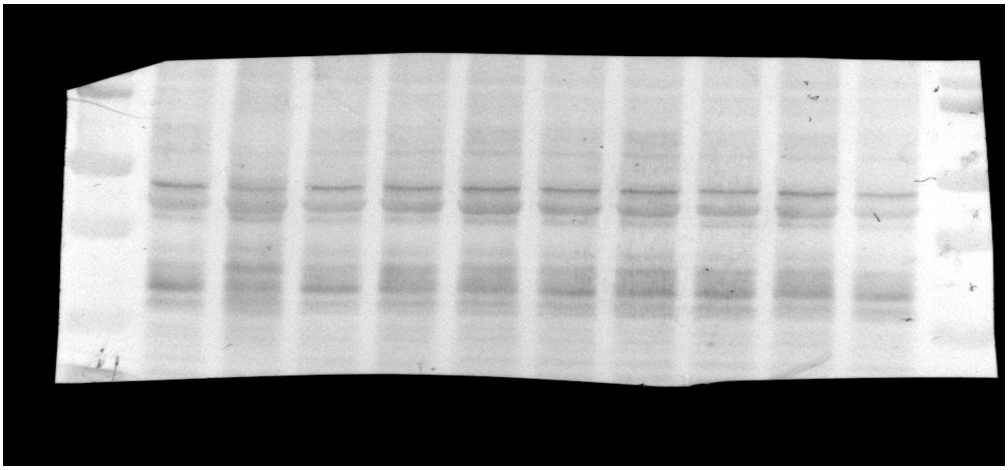

E16
Brain

HIF1α

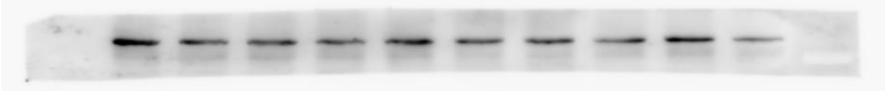

GAPDH

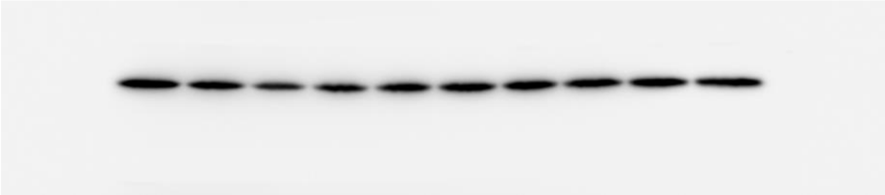

β-Tubulin

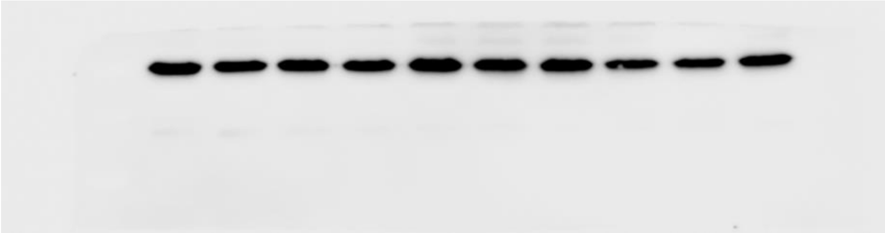

Total protein
(AmidoBlack)

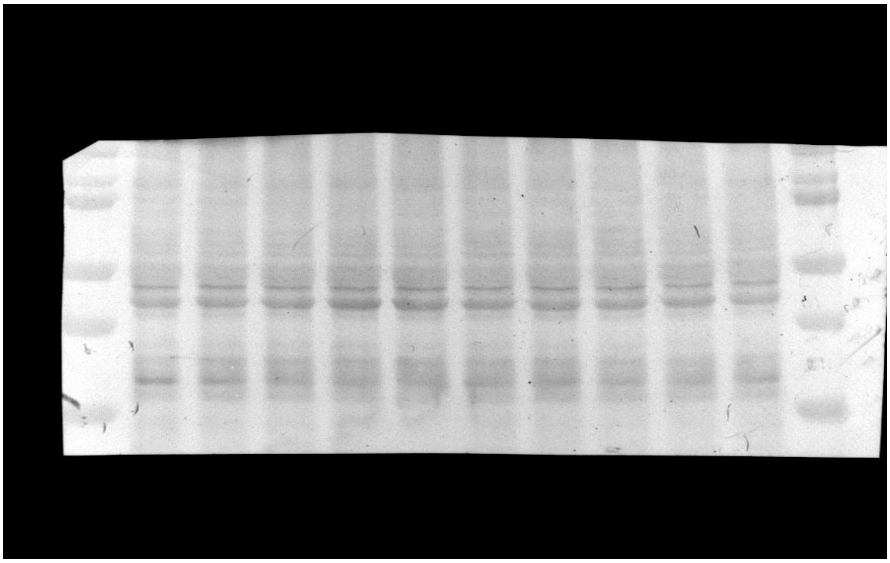

E18
Brain

HIF1α

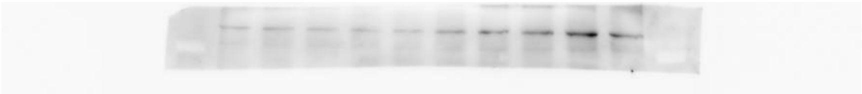

GAPDH

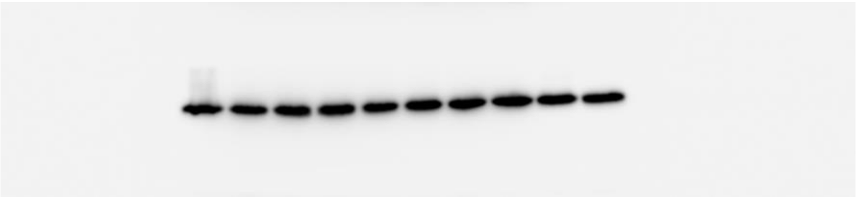

β-Tubulin

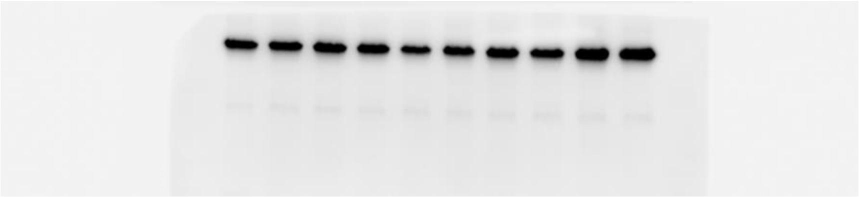

Total protein
(AmidoBlack)

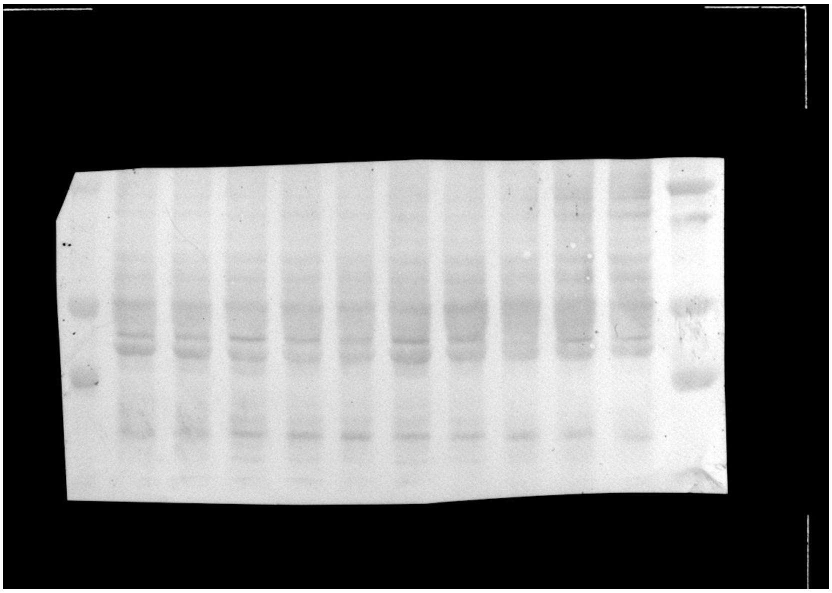

E20
Brain

HIF1α

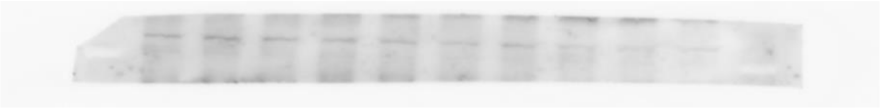

GAPDH

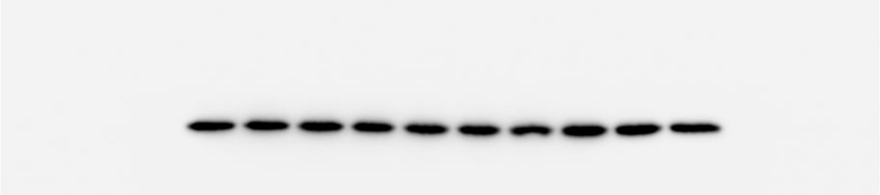

β-Tubulin

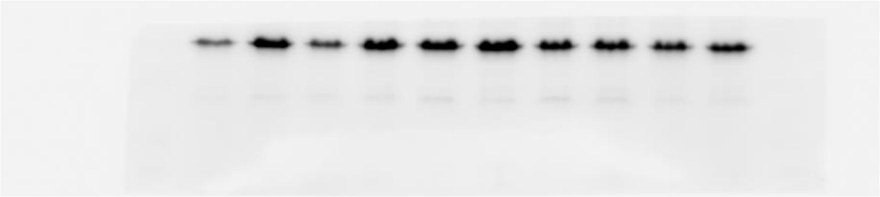

Total protein (AmidoBlack)

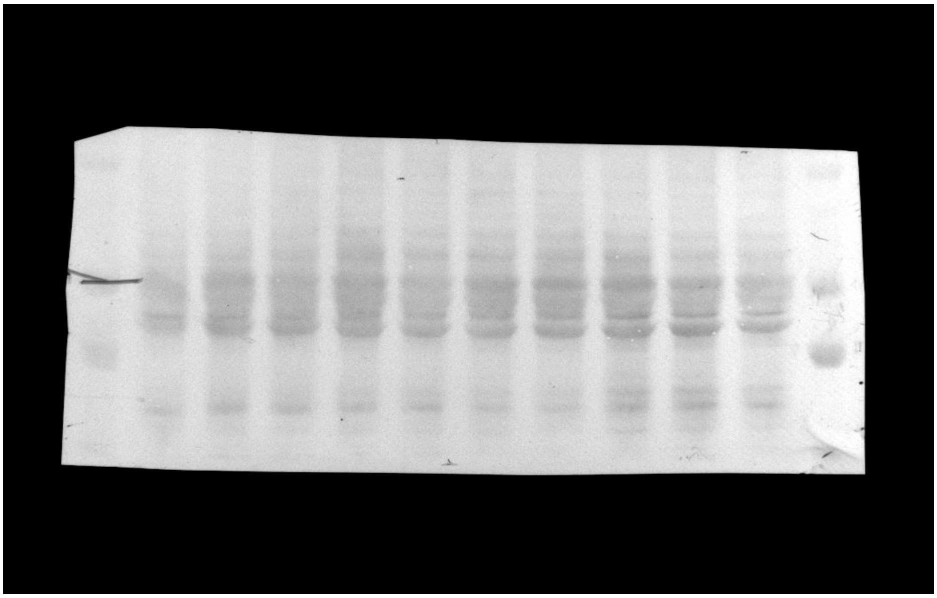

HPC

HIF1α

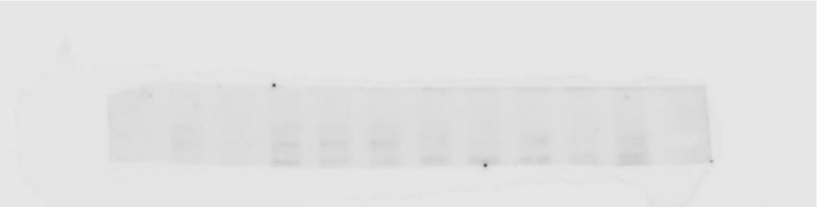

LDHA

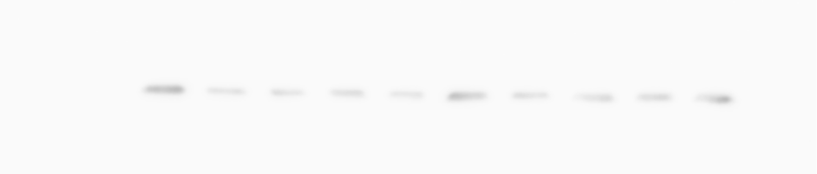

G6PD

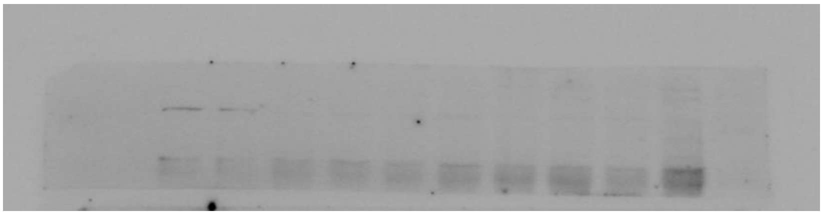

GAPDH

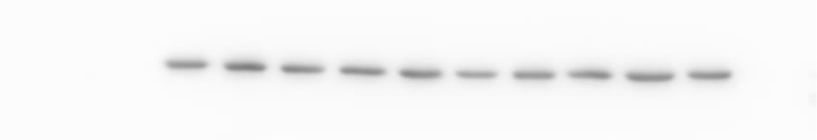

β-Tubulin

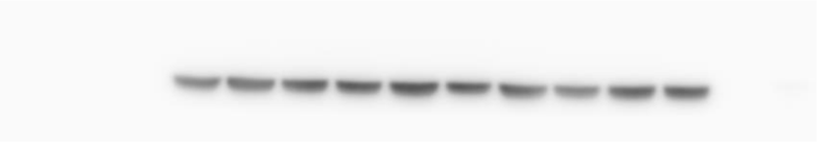

Total protein (AmidoBlack)

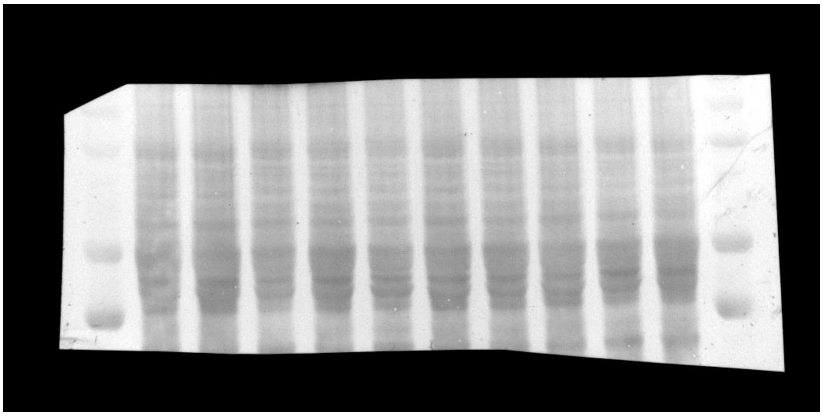

PFC

HIF1α

LDHA

G6PD

GAPDH

β-Tubulin

Total protein (AmidoBlack)

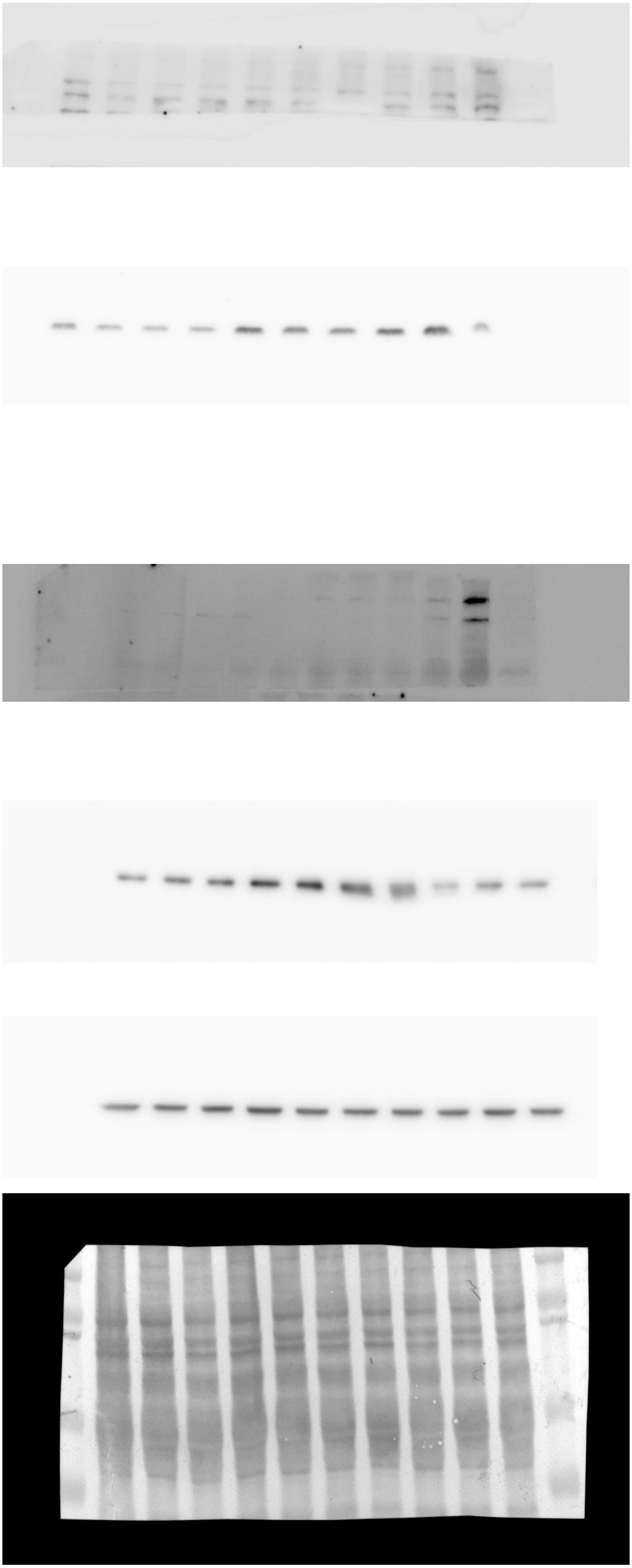

AMG

HIF1α

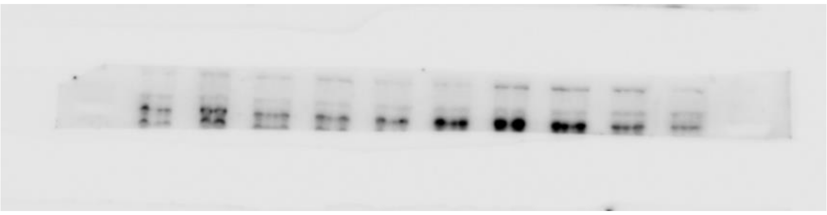

LDHA

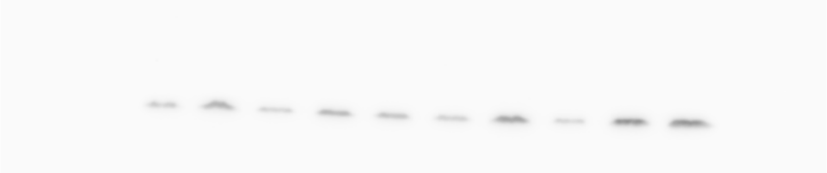

G6PD

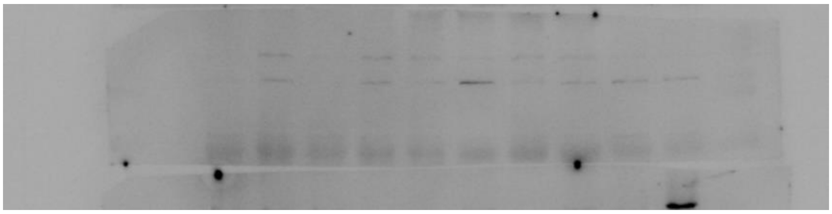

GAPDH

β-Tubulin

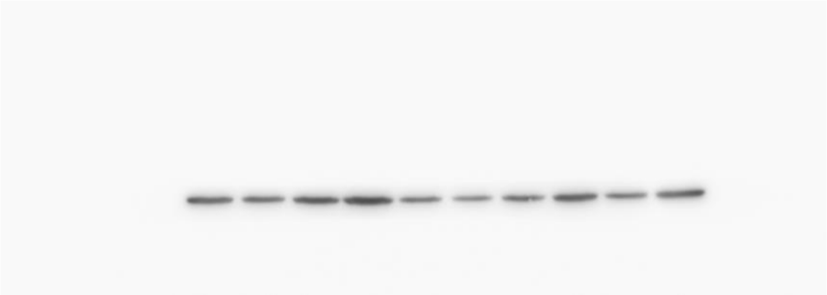

Total protein (AmidoBlack)

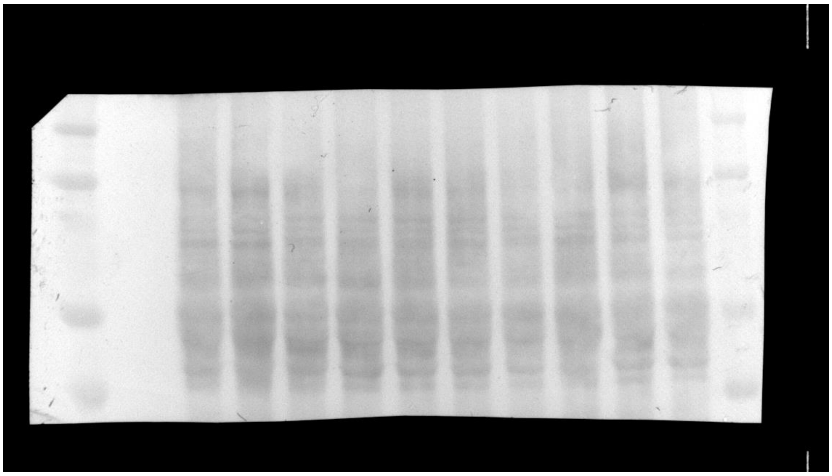

NAcc

HIF1α

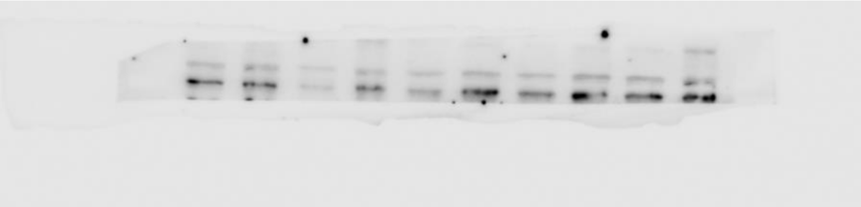

LDHA

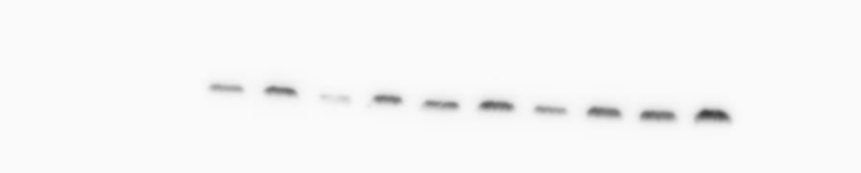

G6PD

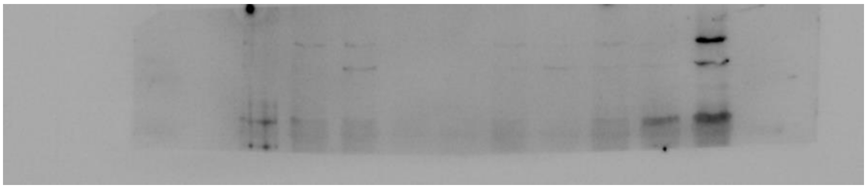

GAPDH

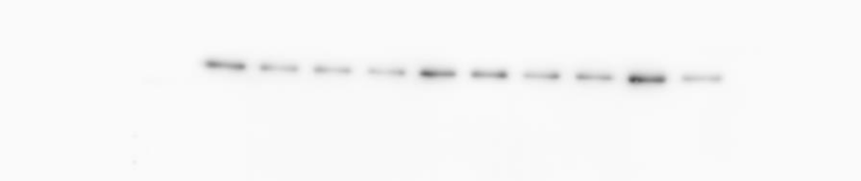

β-Tubulin

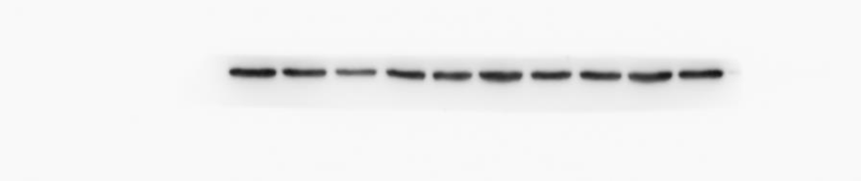

Total protein
(AmidoBlack)

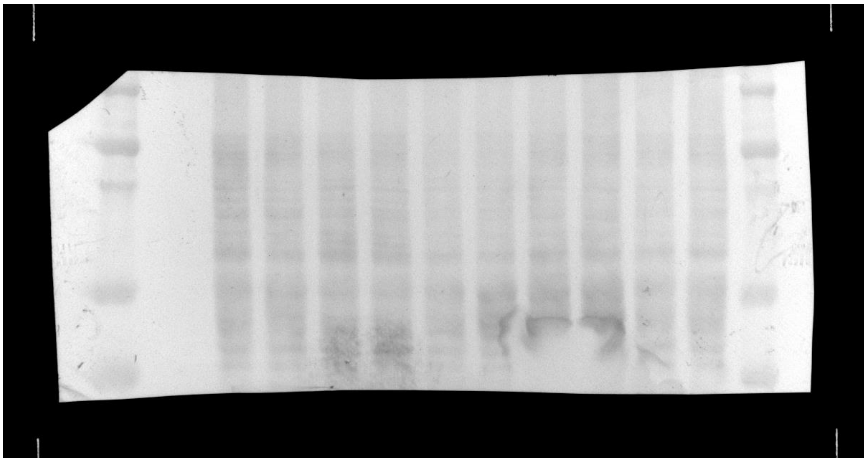

VTA

HIF1α

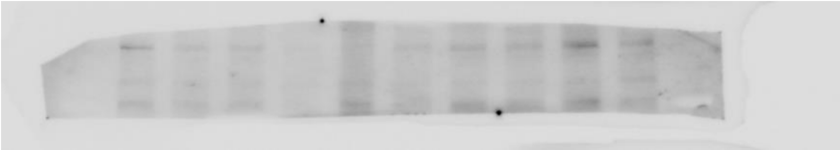

LDHA

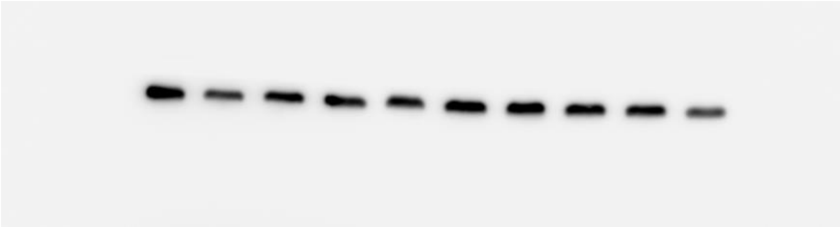

G6PD

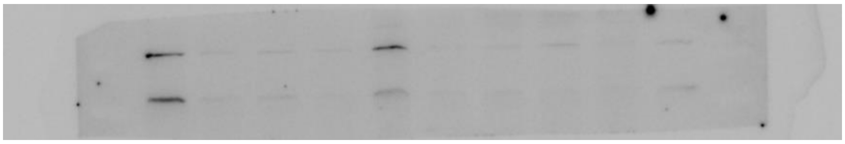

GAPDH

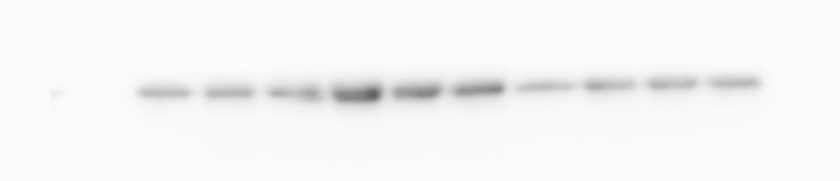

β-Tubulin

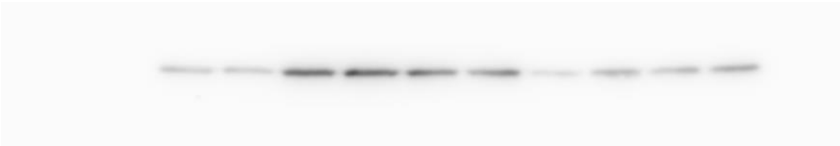

Total protein (AmidoBlack)

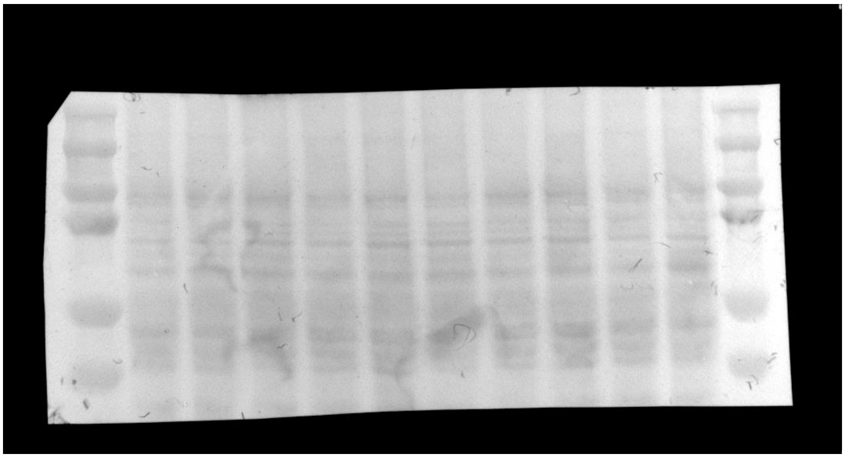

RN

HIF1α

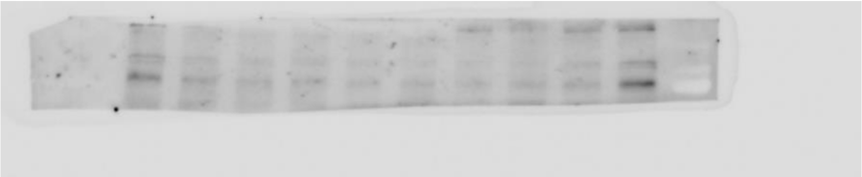

LDHA

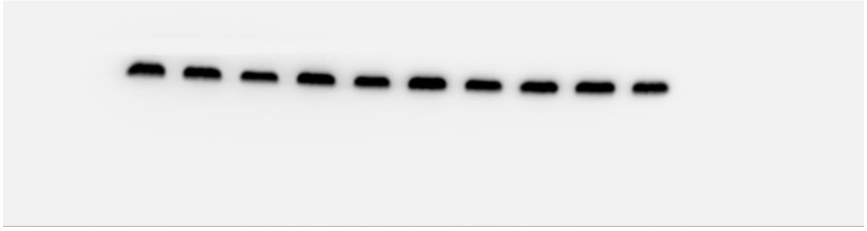

G6PD

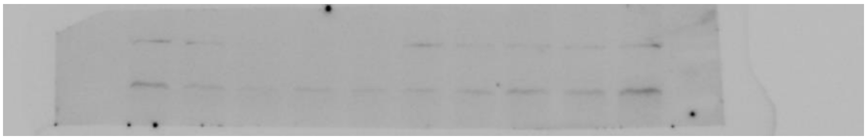

GAPDH

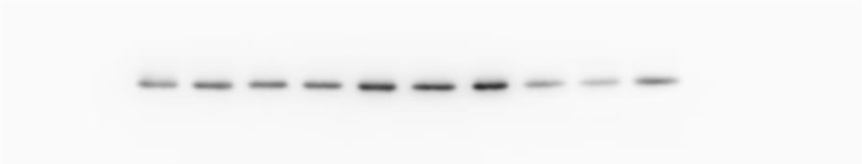

β-Tubulin

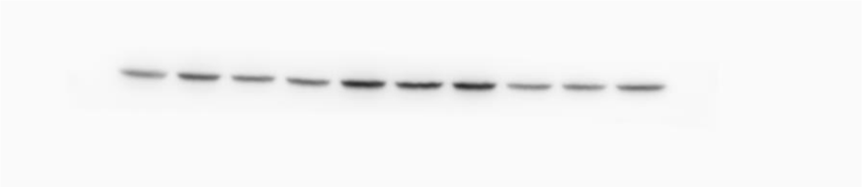

Total protein
(AmidoBlack)

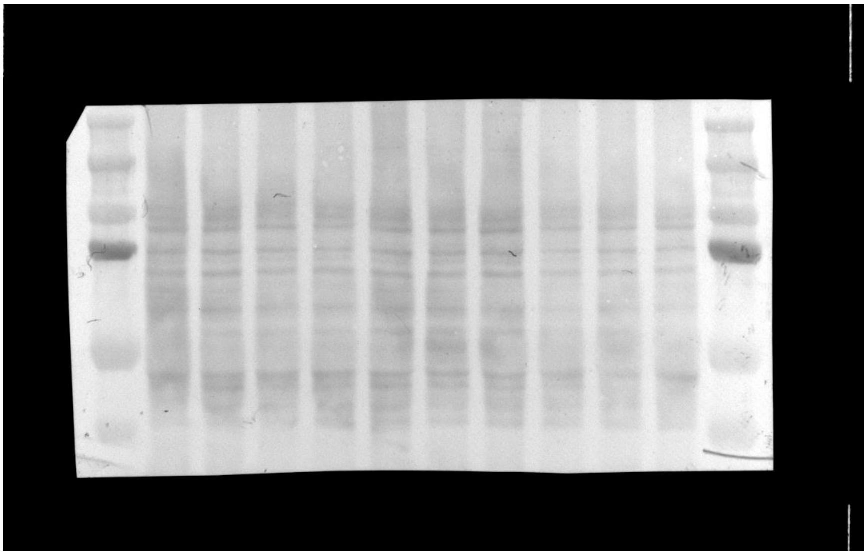

Supplement: Supplementary file 1 [file ijms-27-03421-s001.zip › ijms-4252390-supplementary.pdf]
